# Integrated molecular, immunoinformatic, and structural analysis reveals emerging antigenic divergence of Foot-and-mouth disease virus serotype O during the 2022–2023 outbreaks in Indonesia

**DOI:** 10.14202/vetworld.2026.888-904

**Published:** 2026-03-12

**Authors:** Rahma Isartina Anwar, Rika Indri Astuti, Ni Luh Putu Ika Mayasari, Tri Puji Priyatno, Santoso Santoso, Harimurti Nuradji, Aris Tri Wahyudi

**Affiliations:** 1 Division of Microbiology, Department of Biology, Faculty of Mathematics and Natural Sciences, IPB University, Bogor, Indonesia; 2 Research Center for Animal Husbandry, National Research and Innovation Agency, Bogor, Indonesia; 3 Division of Medical Microbiology, Faculty of Veterinary Medicine, IPB University, Bogor, Indonesia; 4 Research Center for Veterinary Research, National Research and Innovation Agency, Bogor, Indonesia

**Keywords:** foot-and-mouth disease virus, immunoinformatics, Indonesia, molecular docking, phylogenetic analysis, serotype O, TLR7 interaction, *VP1* gene

## Abstract

**Background and Aim:**

After more than three decades of freedom from foot-and-mouth disease (FMD), Indonesia experienced widespread outbreaks in 2022–2023, raising major concerns regarding viral evolution and vaccine effectiveness. Foot-and-mouth disease virus (FMDV) serotype O remains the predominant circulating serotype in the region. However, the immunological and structural consequences of recent genetic variation have not been comprehensively evaluated. This study aimed to integrate molecular, immunoinformatic, and structural analyses to characterize FMDV serotype O isolates from West Java and South Sumatra and to assess their implications for antigenicity, immune recognition, and vaccine matching.

**Materials and Methods:**

Clinical epithelial samples were collected from naturally infected cattle during outbreaks in West Java and South Sumatra. Viral RNA was extracted, and the capsid genes *VP1*, *VP2*, and *VP3* were amplified and sequenced. Phylogenetic relationships were inferred using VP1 nucleotide and amino acid sequences. Immunoinformatic analyses were conducted to predict *VP1*-derived T-cell (BoLA-restricted) and B-cell epitopes, followed by in silico evaluation of antigenicity, allergenicity, and toxicity. Structural analyses included prediction of *VP1* ligand-binding pockets and molecular docking between *VP1* and Toll-like receptor 7 (*TLR7*) to explore innate immune recognition.

**Results:**

Sequence comparison revealed reduced identity of *VP1* (98.26–99.05%) and *VP3* (as low as 98.48%) relative to the 2022 Indonesian reference strain. Phylogenetic analysis identified three nucleotide-based clusters and two amino acid–based clusters, indicating intra-country diversification and the emergence of potential micro-lineages. Several amino acid substitutions occurred near known immunogenic regions of *VP1*, resulting in altered T- and B-cell epitope binding profiles in selected isolates. Predicted epitopes were predominantly antigenic and non-toxic, although some showed potential allergenicity. Structural modeling demonstrated variability in *VP1* binding-pocket composition among isolates. Docking analysis revealed favorable *VP1–TLR7* interactions, particularly in selected South Sumatra isolates, suggesting strong innate immune engagement.

**Conclusion:**

This integrated molecular–immunoinformatic–structural analysis demonstrates that newly circulating Indonesian FMDV serotype O isolates exhibit genetic, antigenic, and structural divergence that may reduce current vaccine matching. Continuous molecular surveillance and regionally adapted vaccine design are therefore essential to maintain effective FMD control in Indonesia.

## INTRODUCTION

Foot-and-mouth disease (FMD) is a highly contagious viral disease affecting cloven-hoofed livestock, including cattle, goats, sheep, and pigs, and it also infects wild ungulates, particularly species at risk of extinction [1]. Caused by an aphthovirus, FMD spreads rapidly through direct contact with infected animals or via contaminated fomites. Clinically, the disease is characterized by fever, vesicular lesions in the oral cavity and feet, lameness, and reduced milk yield, leading to substantial economic losses for livestock producers. Vaccination programs combined with strict biosecurity measures are therefore essential for effective disease prevention and control. Although FMD has been eradicated in several regions, including Australia, Canada, Greenland, Mexico, and New Zealand, it remains a major transboundary animal disease threat in many parts of Africa, Asia, and South America.

Foot-and-mouth disease virus (FMDV) is a positive-sense single-stranded RNA virus with a genome of approximately 8400 nucleotides. Viral particles are spherical, with diameters ranging from 25 to 30 nm, and consist of an RNA genome enclosed within a protein capsid. The capsid is composed of 60 capsomeres, each containing four structural polypeptides: VP1, VP2, VP3, and VP4. The proteins VP1, VP2, and VP3 are exposed on the viral surface, whereas *VP4* is located internally [2]. The surface-exposed proteins (VP1, VP2, and VP3) constitute the major antigenic components of the virus [3]. The VP1 protein contains two key immunogenic regions, namely the G–H loop (amino acid positions 141–160) and the C-terminus (positions 200–213). The G–H loop harbors the arginine–glycine–aspartic acid (RGD) motif, which is essential for viral attachment to host cells through integrin receptors [4]. Owing to its critical role in host cell attachment, immune recognition, and serotype specificity, the *VP1* coding region is widely used for genetic characterization of FMDV strains. Sequences of *VP1* are also routinely employed in phylogenetic analyses to investigate epidemiological relationships and to trace the origin and spread of virus strains during outbreaks [5].

In 2022, FMD outbreaks continued to affect several Asian countries, including Cambodia, China, peninsular Malaysia, Indonesia, Thailand, and Vietnam, whereas no new outbreaks were reported in Lao PDR, Mongolia, or Myanmar. The importation of cattle and meat products markedly increases the risk of FMD transmission, as the virus can disseminate rapidly through livestock movement between regions. After more than 30 years of disease freedom, Indonesia experienced a major re-emergence of FMD in 2022. The outbreak initially reappeared in Gresik, East Java, and subsequently spread swiftly across multiple provinces, including West Java, Bali, West Kalimantan, Central Kalimantan, East Kalimantan, and West Nusa Tenggara, ultimately affecting almost all provinces on Sumatra.

Phylogenetic analysis reported in a previous study [6] indicated that the virus responsible for the 2022 outbreak originated from Gresik and Banjarnegara in East Java Province. Clinical samples analyzed in the present study were obtained from FMD outbreaks in Indonesia during 2022–2023. Indonesia is now considered endemic for FMD, and the introduction of novel viral lineages into Southeast Asia has been increasingly reported [6].

Despite extensive molecular surveillance of FMDV in Southeast Asia, most studies conducted after the 2022 re-emergence in Indonesia have primarily focused on partial genomic characterization and lineage assignment based on *VP1* phylogeny. While these analyses have clarified the geographic origin and spread of circulating strains, they provide limited insight into the functional immunological consequences of emerging genetic variation. In particular, there is a lack of integrated evidence linking sequence divergence in *VP1*, *VP2*, and *VP3* with changes in T- and B-cell epitope profiles, antigenicity, and innate immune recognition. Moreover, structural features such as ligand-binding pocket variability and host receptor interactions, especially involving *TLR7*, remain largely unexplored for Indonesian FMDV isolates. This gap hampers the ability to assess potential vaccine mismatch, host-specific immune responsiveness, and the emergence of micro-lineages that may compromise current vaccination strategies. Consequently, a comprehensive, multi-layered analysis combining molecular, immunoinformatic, and structural approaches is needed to better understand the evolving antigenic landscape of FMDV in Indonesia.

The present study aimed to comprehensively characterize FMDV serotype O isolates collected during the 2022–2023 outbreaks in Indonesia using an integrated molecular, immunoinformatic, and structural framework. Specifically, this study sought to (i) determine the genetic diversity and phylogenetic relationships of circulating isolates based on *VP1*, *VP2*, and *VP3* sequences, (ii) predict and compare *VP1*-derived T-cell and B-cell epitopes to identify potential antigenic shifts relative to recent reference strains, (iii) evaluate the antigenicity, allergenicity, and toxicity of predicted epitopes using in silico approaches, (iv) analyze variation in *VP1* ligand-binding pockets that may influence antigen–antibody interactions, and (v) assess the interaction potential between *VP1* and *TLR7* through molecular docking. Through this integrated approach, the study aimed to generate functional immunogenomic evidence relevant to vaccine matching and to support adaptive FMD control strategies in Indonesia.

## MATERIALS AND METHODS

### Ethical approval

All animal procedures were conducted in accordance with Indonesia’s national guidelines for animal welfare and biosafety for transboundary animal diseases. The study protocol, including field sampling, animal handling, and laboratory processing of infectious materials, was reviewed and approved by the Ethical Clearance Committee of the National Research and Innovation Agency (BRIN), Indonesia (approval no. 065/KE.02/SK/10/2022, October 6, 2022). Only naturally infected animals identified during the FMD outbreak were included; no experimental infection, disease induction, or invasive procedures were performed. Oropharyngeal epithelium and lesion scrapings were collected by licensed veterinarians and trained animal health officers following World Organisation for Animal Health (WOAH) guidelines for safe FMD sampling. Animals were handled using minimal-restraint techniques without sedation to avoid pain or distress. Farmers or animal owners provided prior informed consent, and all activities were supervised by the District Livestock Services in West Java and South Sumatra. Sample collection was conducted using appropriate personal protective equipment, and transport complied with national regulations for infectious substances. Laboratory work, including RNA extraction, cDNA synthesis, polymerase chain reaction (PCR) amplification, and sample storage, was performed in a BSL-2 facility with Class II biological safety cabinets. Waste materials were decontaminated by autoclaving or chemical disinfection. The study adhered to Animal Research: Reporting of *In Vivo* Experiments 2.0, the WOAH Terrestrial Manual (Chapter 3.1.8), and the Three Rs principles.

### Study period and location

**Figure 1:**
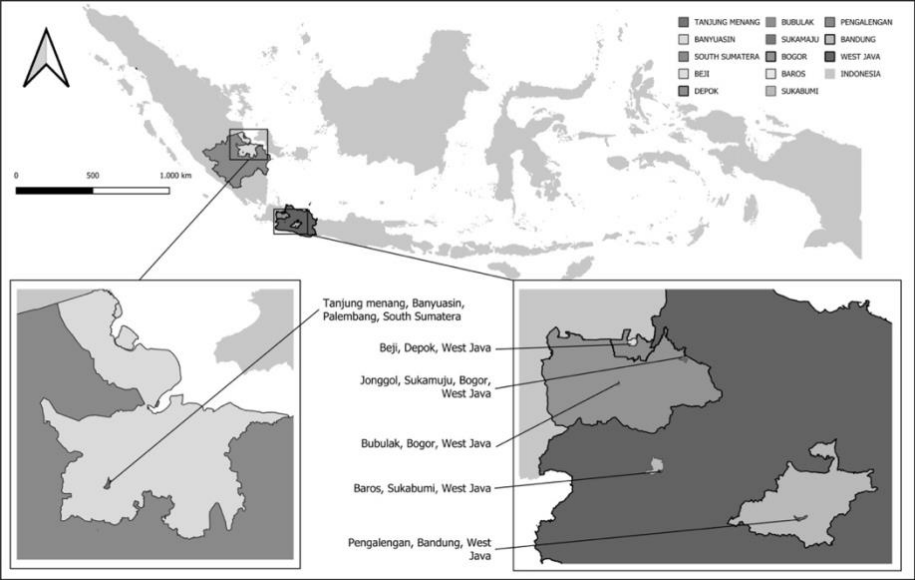
The geographical location of the sample collection areas in West Java and South Sumatra.

Samples were obtained from suspected FMD cases based on clinical signs (hypersalivation and vesicular lesions of the oral cavity and feet) in West Java and South Sumatra, Indonesia, during 2022–2023 (Figure 1). Sampling was conducted on smallholder cattle farms raising local breeds (*Bos indicus* × *Bos taurus*; Peranakan Ongole, crossbred, and Bali), with herd sizes of approximately 50–100 animals.

### Sample collection and transportation

**Figure 2:**
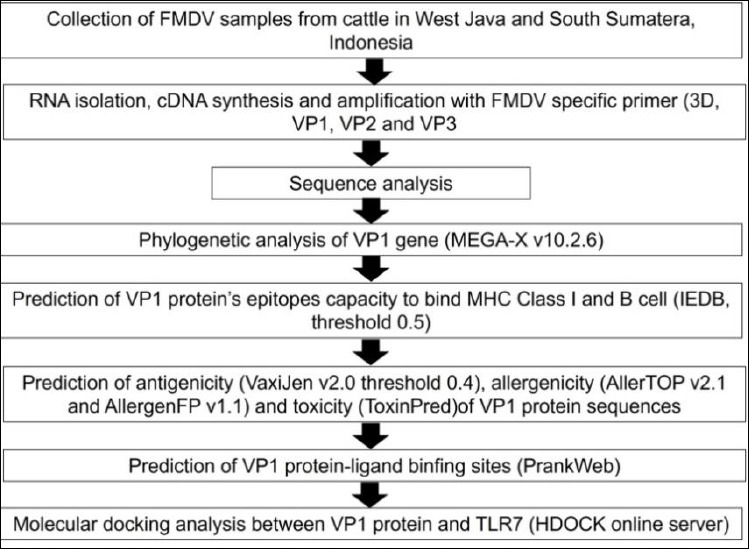
The methodology schematic used in this study.

The methodology used in this study is presented in Figure 2. Animals with clinical scores of 2 and 3 (moderate and severe, respectively) presenting vesicles and ulceration of the oral cavity and interdigital spaces of the feet, hypersalivation, and lameness were selected. A total of 28 clinical samples (oral and foot epithelium) were collected and preserved in RNA Shield (Zymo Research, Irvine, CA, USA) (catalog no. R1100-50). The samples were transported in insulated containers with ice packs at approximately 4°C, with temperature validation using temperature logs, over 48–72 h. RNA Shield (Zymo Research) was used to protect nucleic acids at ambient temperature for up to 7 days. All samples were stored at −20°C in the laboratory for further analysis.

### RNA extraction and cDNA synthesis

Total RNA extraction was performed according to the manufacturer’s instructions using the Quick-RNA Miniprep Kit (Zymo Research) (catalog no. R1054), with a total RNA elution volume of 30 µL. RNA quality and concentration were evaluated using a NanoDrop spectrophotometer, and RNA with an A260/280 ratio of 1.8–2.0 was selected for further processing. cDNA synthesis was performed using the RevertAid cDNA Synthesis Kit (Thermo Fisher Scientific, Waltham, MA, USA) (catalog no. K1622) to generate complementary DNA. Negative controls (RNase/DNase-free water) were included to exclude contamination. The reaction setup involved incubation at 65°C for 5 min, followed by cDNA synthesis at 25°C for 5 min, 42°C for 60 min, and enzyme inactivation at 70°C for 5 min.

### PCR amplification and capsid gene analysis

The cDNA was amplified by conventional PCR using DreamTaq PCR Master Mix (Thermo Fisher Scientific) (catalog no. K1081) with 3D primers for FMD diagnosis [7]. PCR conditions included initial denaturation at 95°C for 5 min, followed by 35 cycles of denaturation at 95°C for 10 s, annealing at 60°C for 20 s, and extension at 72°C for 30 s, with a final extension at 72°C for 5 min. The capsid genes in positive samples were analyzed using primers designed based on the FMDV serotype O reference sequence (MN095357.1) (Table 1). Gene-specific annealing temperatures were applied for *VP1*, *VP2*, and *VP3*. PCR amplification was performed in triplicate, and amplicons were pooled. RNase/DNase-free water was used as a negative control. Amplicons were confirmed on 1.5% agarose gels and purified using a PCR Clean-Up Kit (Geneaid, New Taipei City, Taiwan) (catalog no. DFC100). Purified products were sequenced using Sanger sequencing (BigDye Terminator v3.1, 1st BASE), and sequence identity was confirmed using BLAST against the NCBI GenBank database.

**Table 1:** Primers used for foot-and-mouth disease virus capsid diagnosis and analysis.

Gene	Primer sequence (5′–3′)
3D	Forward: ACTGGGTTTTACAAACCTGTGA
	Reverse: GCGAGTCCTGCCACGGA
VP1	Forward: CTTCTCCGACGTTAGGTC
	Reverse: GTCACGTGCTTTGAGCTG
VP2	Forward: CTTCTCCGACGTTAGGTC
	Reverse: GTCACGTGCTTTGAGCTG
VP3	Forward: CTTCTCCGACGTTAGGTC
	Reverse: GTCACGTGCTTTGAGCTG

### Sequence quality assessment and data submission

Sequence data were analyzed using MEGA-X v10.2.6. Low-quality regions were trimmed based on chromatogram inspection, Phred quality scores <Q20 were excluded, and ambiguous terminal bases were removed. Sequences were aligned using the MUSCLE algorithm. All complete *VP1* sequences were deposited in GenBank on October 2, 2025 (accession nos. PX406137–PX406155).

### Phylogenetic analysis of *VP1*

Aligned *VP1* nucleotide and amino acid sequences were analyzed using MEGA-X v10.2.6 and compared with reference FMDV isolates [8]. Phylogenetic trees were constructed using the maximum likelihood method under the generalized time-reversible model with the Kimura 2-parameter substitution model. Bootstrap analysis was conducted with 1000 replicates, and gaps were treated using partial deletion.

### Epitope prediction for MHC class I and B cells

Translated *VP1* amino acid sequences were analyzed using IEDB tools to predict MHC class I–binding epitopes employing NetMHCpan 4.1 EL [9]. BoLA alleles were prioritized. Linear B-cell epitope prediction was performed using IEDB B-cell tools (threshold 0.5) [10]. Experimentally validated high- and low-immunogenic peptides were used as positive and negative controls, respectively.

### Antigenicity, allergenicity, and toxicity assessment

Antigenicity was evaluated using VaxiJen v2.0 [11]. Allergenicity was assessed using AllerTOP v2.1 and AllergenFP v1.1 [12, 13]. Toxicity prediction was conducted using ToxinPred [14]. Predicted epitopes were benchmarked against experimentally validated epitopes.

### Prediction of *VP1* ligand-binding sites

Ligand-binding site prediction and 3D visualization were performed using PrankWeb [15]. *VP1* structural models were generated using Swiss-Model and assessed using standard quality parameters. Binding pockets were ranked based on calibrated probability scores.

### Molecular docking between *VP1* and *TLR7*

Molecular docking between *VP1* and *TLR7* was performed using the HDOCK server [16]. Docking scores and confidence scores were used to evaluate binding probability, and docking complexes were visualized using Swiss-PdbViewer v4.1.0.

## RESULTS

### Detection and sequencing of FMDV serotype O capsid genes

Local FMDV serotype O strains were obtained from naturally infected cattle in South Sumatra and West Java during the 2022–2023 outbreak period (Table 2). PCR amplification was performed using serotype O–specific primers targeting the capsid gene coding regions *VP1*, *VP2*, and *VP3*. Sequencing quality was evaluated using Phred quality scores, and only reads meeting a Q20 threshold were included for downstream analysis. Comparative sequence analysis demonstrated that the *VP1*, *VP2*, and *VP3* genes showed reduced nucleotide identity relative to the most recent Indonesian reference strain, FMDV serotype O isolate ISA/1/2022 (Table 3). For *VP1*, samples Depok 2, Palembang 8, Sukabumi 1, and Sukabumi 2 exhibited the lowest sequence identity (98%). A similar pattern was observed for *VP2*, with Sukabumi 1 and Sukabumi 2 also showing 98% identity. In contrast, *VP3* identity decreased to 98% in most samples, except for Depok 1 and Bogor 1, which retained higher similarity (99%).

**Table 2:** Foot-and-mouth disease virus diagnosis and *VP1*, *VP2*, and *VP3* gene analysis of collected samples.

Sample code	City/Province	*3D*	*VP1*	*VP2*	*VP3*
Bandung 1	Bandung, West Java, Indonesia	+	+	+	+
Bandung 2	Bandung, West Java, Indonesia	−	−	−	−
Bandung 3	Bandung, West Java, Indonesia	+	+	+	+
Bandung 4	Bandung, West Java, Indonesia	+	+	+	+
Bandung 5	Bandung, West Java, Indonesia	+	+	+	+
Bandung 6	Bandung, West Java, Indonesia	−	−	−	−
Bandung 7	Bandung, West Java, Indonesia	−	−	−	−
Bandung 8	Bandung, West Java, Indonesia	+	+	+	+
Bandung 9	Bandung, West Java, Indonesia	+	+	+	+
Palembang 1	Palembang, South Sumatra, Indonesia	+	+	+	+
Palembang 2	Palembang, South Sumatra, Indonesia	+	+	+	+
Palembang 3	Palembang, South Sumatra, Indonesia	+	+	+	+
Palembang 4	Palembang, South Sumatra, Indonesia	+	+	+	+
Palembang 5	Palembang, South Sumatra, Indonesia	−	−	−	−
Palembang 6	Palembang, South Sumatra, Indonesia	+	+	+	+
Palembang 7	Palembang, South Sumatra, Indonesia	+	+	+	+
Palembang 8	Palembang, South Sumatra, Indonesia	+	+	+	+
Palembang 9	Palembang, South Sumatra, Indonesia	+	+	+	+
Bogor 1	Bogor, West Java, Indonesia	+	+	+	+
Bogor 2	Bogor, West Java, Indonesia	−	−	−	−
Bogor 3	Bogor, West Java, Indonesia	−	−	−	−
Bogor 4	Bogor, West Java, Indonesia	−	−	−	−
Bogor 5	Bogor, West Java, Indonesia	−	−	−	−
Bogor 6	Bogor, West Java, Indonesia	−	−	−	−
Depok 1	Depok, West Java, Indonesia	+	+	+	+
Depok 2	Depok, West Java, Indonesia	+	+	+	+
Sukabumi 1	Sukabumi, West Java, Indonesia	+	+	+	+
Sukabumi 2	Sukabumi, West Java, Indonesia	+	+	+	+

+ detected, − undetected

**Table 3:** Comparison of gene sequence similarity between collected samples and recent FMDV O isolate ISA/1/2022.

Sample	*VP1*	*VP2*	*VP3*
Bandung 1	99.68%	99.85%	98.64%
Bandung 3	99.68%	99.85%	98.64%
Bandung 4	99.68%	99.85%	98.92%
Bandung 5	99.68%	99.85%	98.92%
Bandung 8	99.68%	99.85%	98.92%
Bandung 9	99.21%	99.38%	98.92%
Palembang 1	99.05%	99.85%	98.48%
Palembang 2	99.05%	99.85%	98.48%
Palembang 3	99.05%	99.85%	98.48%
Palembang 4	99.05%	99.22%	98.93%
Palembang 6	99.05%	99.85%	98.48%
Palembang 7	99.05%	99.19%	98.93%
Palembang 8	98.58%	99.85%	98.48%
Palembang 9	99.05%	99.85%	98.48%
Bogor 1	99.68%	99.69%	99.53%
Depok 1	99.68%	99.69%	99.53%
Depok 2	98.26%	99.03%	98.59%
Sukabumi 1	98.42%	98.77%	98.73%
Sukabumi 2	98.42%	98.77%	98.73%

FMDV = Foot-and-mouth disease virus, ISA = Indonesian isolate.

### Phylogenetic analysis of *VP1*

**Figure 3:**
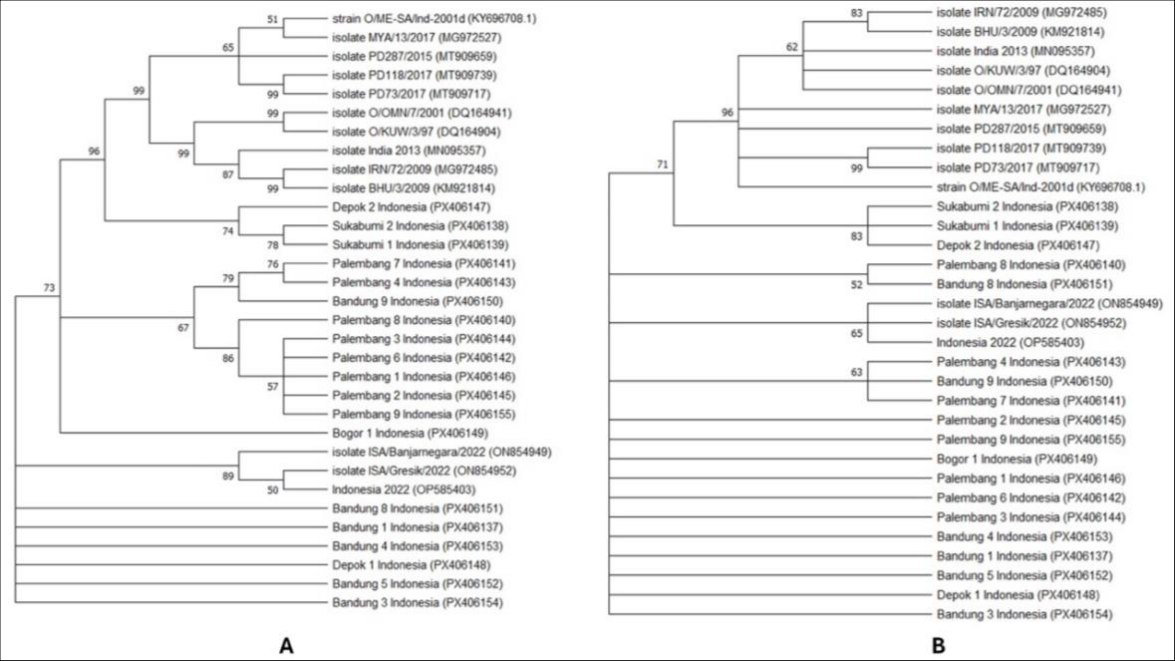
Phylogenetic tree using MEGA-X v10.2.6, the maximum likelihood method based on full *VP1* gene of 19 FMDV isolates sequences from collection in the study with additional 12 sequences of FMDV serotype O from GenBank database. Phylogeny testing using the bootstrap method with 1000 replicates and Kimura 2 parameter. Substitution type mode using (A) nucleic acid and (B) amino acid.

Phylogenetic reconstruction based on nucleotide substitution models classified the recent FMDV serotype O isolates into three principal clusters. Cluster I comprised Depok 2, Sukabumi 1, Sukabumi 2, and reference FMDV serotype O strains from the database (O/ME-SA/Ind-2001d, Myanmar, India, Oman, Kuwait, Iran, and Bhutan). Cluster II included Bandung 9 and Palembang 1, 2, 3, 4, 6, 7, and 8. Cluster III consisted of Bogor 1, Bandung 1, 3, 4, 5, 8, and Depok 1, which showed close genetic relatedness to Indonesian reference isolates ISA/Gresik/2022 and ISA/Banjarnegara/2022. In contrast, phylogenetic analysis based on amino acid substitutions resolved only two main clusters: Cluster I included Depok 2, Sukabumi 1, Sukabumi 2, and related database isolates, whereas Cluster II comprised the remaining samples together with ISA/Gresik/2022 and ISA/Banjarnegara/2022 (Figure 3).

Comparative analysis of *VP1* sequences identified multiple nucleotide variations relative to Indonesian and other serotype O reference sequences, particularly at positions 10, 37, 84, 102, 103, 171, 219, 225, 386, 408, 451, 459, 462, 465, 515, 526, 582, 589, 615, and 627 (Table 4). These substitutions resulted in amino acid changes at positions 34, 113, 129, 172, and 197 (Table 5).

**Table 4:** Nucleic acid variation in the *VP1* gene between collected samples and ISA/Indonesia 2022 isolates.

Position of nucleic acid	ISA/Indonesia 2022	Bandung 1, 3, 4	Bandung 5	Bandung 8	Bandung 9	Sukabumi 1, 2	Bogor 1	Depok 2	Depok 1	Palembang 1, 2, 3, 6, 9	Palembang 4, 7	Palembang 8
10	G	A	A	A	A	A	A	A	A	A	A	A
37	G	G	G	G	G	A	G	A	G	G	G	G
84	G	G	G	G	G	G	G	G	G	G	A	G
102	T	T	T	T	T	C	T	C	T	T	T	T
103	A	A	A	A	G	A	A	A	A	A	G	A
171	C	C	C	C	C	T	C	T	C	C	C	C
219	C	C	C	C	C	T	C	T	C	C	C	C
225	C	C	C	C	C	T	C	T	C	C	C	C
386	C	C	C	C	T	T	T	T	C	T	T	T
408	C	C	C	C	C	C	C	T	C	C	C	C
451	T	C	C	C	C	T	T	T	T	C	C	C
459	G	G	G	C	G	G	G	G	G	G	G	T
462	G	G	G	G	G	G	G	G	G	G	G	T
465	A	A	A	A	A	A	A	A	A	G	A	G
515	G	G	G	G	G	A	G	A	G	G	G	G
526	T	T	T	T	T	T	T	T	T	C	T	C
582	T	T	C	T	T	T	T	T	T	T	T	T
589	G	G	G	G	G	G	G	G	G	G	G	A
615	C	C	C	C	C	T	C	T	C	C	C	C
627	C	C	C	C	C	T	C	T	C	C	C	C

ISA = Indonesian isolate.

**Table 5:** Amino acid variation in VP1 protein between collected samples and ISA/Indonesia 2022 isolate.

Amino acid position	ISA/Indonesia 2022	Bandung 1, 3, 4, 5, 8, 9	Sukabumi 1, 2	Bogor 1	Depok 2	Depok 1	Palembang 1, 2, 3, 4, 6, 7, 9	Palembang 8
4	A	T	T	T	T	T	T	T
13	A	A	T	A	T	A	A	A
129	A	A	V	V	V	A	V	V
172	R	R	Q	R	Q	R	R	R
197	E	E	E	E	E	E	E	K

ISA = Indonesian isolate

### Epitope prediction and immunological profiling of *VP1*

Based on MHC class I–binding predictions, the VP1 protein yielded eight peptides capable of binding T-cell epitopes associated with BoLA-1, BoLA-2, BoLA-3, BoLA-4, BoLA-5, BoLA-6, BoLA-T5, and BoLA-T7 alleles. Compared with Ind/2022, ISA/Banjarnegara/Gresik, and other FMDV serotype O isolates, samples from Bandung 1, 3, 4, 5, 8, 9, and Palembang 8 exhibited distinct T-cell epitope binding profiles. Samples Depok 2 and Sukabumi 1 and 2 differed from Ind/2022 and ISA/Banjarnegara/Gresik only at amino acid positions 4 (alanine) and 13 (tyrosine), localized within residues 2–15 of *VP1* corresponding to the BoLA-T7 allele. No epitope differences were observed for other alleles. All predicted peptides were antigenic and non-toxic, although several were classified as probable allergens (Table 6).

**Table 6:** Prediction of *VP1* binding to T cells among collected samples and database.

BoLA allele (position)	Peptide (aa position)	Peptide sequence	Databases and code samples	Antigenicity score	Antigenicity	Allergenicity	Toxicity	Percentile rank
BoLA-1:00901 (69–82)	69–82	ATYYFADLEVAVKH	Strain O/ME-SA/Ind, MYA/13/2017, PD287/2015, PD73/2017, IRN/72/2009, BHU/3/2009, O/OMN/7/2001, O/KUW/3/97, INDIA 2013, ISA/GRESIK/2022, ISA/BANJARNEGARA/2022, INDONESIA 2022, Bandung 1, 3, 4, 5, 8, 9, Depok 1, 2, Bogor 1, Sukabumi 1, 2, Palembang 1, 2, 3, 4, 6, 7, 8, 9	0.9473	Antigen	Non-allergen	Non-toxin	2.6
		ATYYFAELEVAVKH	PD118/2017 (India)	0.7559	Antigen	Allergen	Non-toxin	2.5
BoLA-2:00501 (38–51)	38–51	RFVKVTPKDQINVL	Strain O/ME-SA/Ind, MYA/13/2017, PD287/2015, PD118/2017, PD73/2017, IRN/72/2009, BHU/3/2009, O/OMN/7/2001, O/KUW/3/97, INDIA 2013, ISA/GRESIK/2022, ISA/BANJARNEGARA/2022, INDONESIA 2022, Bandung 1, 3, 4, 5, 8, 9, Depok 1, 2, Bogor 1, Sukabumi 1, 2, Palembang 1, 2, 3, 4, 6, 7, 8, 9	0.8011	Antigen	Non-allergen	Non-toxin	3.9
BoLA-3:00101 (38–51)	38–51	RFVKVTPKDQINVL	MYA/13/2017, PD287/2015, PD118/2017, PD73/2017, IRN/72/2009, BHU/3/2009, O/OMN/7/2001, O/KUW/3/97, INDIA 2013, ISA/GRESIK/2022, ISA/BANJARNEGARA/2022, INDONESIA 2022, Bandung 1, 3, 4, 5, 8, 9, Depok 1, 2, Bogor 1, Sukabumi 1, 2, Palembang 1, 2, 3, 4, 6, 7, 8, 9	0.8011	Antigen	Non-allergen	Non-toxin	13
BoLA-4:02401 (75–88)	75–88	DLEVAVKHEGNLTW	Strain O/ME-SA/Ind, MYA/13/2017, PD287/2015, PD73/2017, IRN/72/2009, BHU/3/2009, O/OMN/7/2001, O/KUW/3/97, ISA/GRESIK/2022, ISA/BANJARNEGARA/2022, INDONESIA 2022, Bandung 1, 3, 4, 5, 8, 9, Depok 1, 2, Bogor 1, Sukabumi 1, 2, Palembang 1, 2, 3, 4, 6, 7, 8, 9	1.4045	Antigen	Allergen	Non-toxin	4.9
		ELEVAVKHEGNLTW	PD118/2017 (India)	0.7559	Antigen	Allergen	Non-toxin	3.3
		DLEVAVKHKGNLTW	INDIA 2013	1.6457	Antigen	Allergen	Non-toxin	5.4
BoLA-5:00301 (178–191)	178–191	RMKRAETYCPRPLL	Strain O/ME-SA/Ind, MYA/13/2017, PD287/2015, PD118/2017, PD73/2017, IRN/72/2009, BHU/3/2009, O/OMN/7/2001, O/KUW/3/97, INDIA 2013, ISA/GRESIK/2022, ISA/BANJARNEGARA/2022, INDONESIA 2022, Bandung 1, 3, 4, 5, 8, 9, Depok 1, 2, Bogor 1, Sukabumi 1, 2, Palembang 1, 2, 3, 4, 6, 7, 8, 9	0.6731	Antigen	Allergen	Non-toxin	8.9
BoLA-6:01301 (178–191)	178–191	RMKRAETYCPRPLL	Same as above	0.6731	Antigen	Allergen	Non-toxin	0.61
BoLA-T5 (69–82)	69–82	ATYYFADLEVAVKH	Strain O/ME-SA/Ind, MYA/13/2017, PD287/2015, PD73/2017, IRN/72/2009, BHU/3/2009, O/OMN/7/2001, O/KUW/3/97, INDIA 2013, ISA/GRESIK/2022, ISA/BANJARNEGARA/2022, INDONESIA 2022, Bandung 1, 3, 4, 5, 8, 9, Depok 2, Sukabumi 1, 2, Palembang 8	0.9473	Antigen	Non-allergen	Non-toxin	11
		ATYYFAELEVAVKH	PD118/2017 (India)	0.7559	Antigen	Allergen	Non-toxin	8.8
BoLA-T7 (2–15)	2–15	TSTGESADPVTTTV	Strain O/ME-SA/Ind, PD287/2015, PD118/2017, PD73/2017, IRN/72/2009, BHU/3/2009, O/OMN/7/2001, O/KUW/3/97, INDIA 2013, Depok 2, Sukabumi 1, 2	0.8275	Antigen	Allergen	Non-toxin	12
		TSAGESADPVTTTV	MYA/13/2017 (Myanmar)	0.7412	Antigen	Non-allergen	Non-toxin	11
		TSAGESADPVTATV	ISA/GRESIK/2022, ISA/BANJARNEGARA/2022, INDONESIA 2022	0.7250	Antigen	Non-allergen	Non-toxin	13
		TSTGESADPVTATV	Bandung 1, 3, 4, 5, 8, 9, Palembang 8	0.8113	Antigen	Allergen	Non-toxin	14

BoLA = Bovine leukocyte antigen.

B-cell epitope prediction indicated that Sukabumi 1, Sukabumi 2, and Depok 2 differed from ISA/2022 and ISA/Banjarnegara/Gresik by an alanine-to-threonine substitution at position 13 within the 6–30 epitope region. These profiles were otherwise consistent with other FMDV serotype O database isolates. Notably, Palembang 8 exhibited a glutamic acid–to–lysine substitution at position 196 within the 192–205 epitope region, distinguishing it from Ind/2022, ISA/Banjarnegara/Gresik, and other reference isolates. Peptides spanning positions 93–107 were predicted to be non-antigenic across all samples, whereas other regions demonstrated antigenic potential. Several peptides showed predicted allergenicity, while all remained non-toxic (Table 7).

**Table 7:** Prediction of VP1 binding to B cells among collected samples and database.

Position	Peptide sequence	Databases and code samples	Antigenicity prediction score	Antigenicity	Allergenicity	Toxicity
6–30	ESADPVTTTVENYGGETQVQRRQHT	Strain O/ME-SA/Ind, MYA/13/2017 (Myanmar), PD287/2015 (India), PD118/2017, PD73/2017 (India), IRN/72/2009 (Iran), BHU/3/2009 (Bhutan), O/OMN/7/2001 (Oman), O/KUW/3/97 (Kuwait), INDIA 2013, Depok 2, Sukabumi 1, 2	0.5586	Antigen	Allergen	Non-toxin
	ESADPVTATVENYGGETQVQRRQHT	ISA/GRESIK (Indonesia), ISA/BANJARNEGARA (Indonesia), INDONESIA 2022, Bandung 1, 3, 4, 5, 8, 9, Depok 1, Bogor 1, Palembang 1, 2, 3, 4, 6, 7, 8	0.5596	Antigen	Allergen	Non-toxin
41–58	KVTPKDQINVLDLMQTPA	Strain O/ME-SA/Ind, MYA/13/2017 (Myanmar), PD287/2015 (India), PD118/2017, PD73/2017 (India), IRN/72/2009 (Iran), BHU/3/2009 (Bhutan), O/KUW/3/97 (Kuwait), INDIA 2013, ISA/GRESIK (Indonesia), ISA/BANJARNEGARA (Indonesia), INDONESIA 2022, Depok 1, 2, Sukabumi 1, 2, Bandung 1, 3, 4, 5, 8, 9, Bogor 1, Palembang 1, 2, 3, 4, 6, 7, 8	0.8708	Antigen	Non-allergen	Non-toxin
	KVTPKDQINVLDLMKTPA	O/OMN/7/2001 (Oman)	0.7528	Antigen	Non-allergen	Non-toxin
93–107	APETALDNTTNPTAY	Strain O/ME-SA/Ind, O/KUW/3/97 (Kuwait)	0.2242	Non-antigen	Allergen	Non-toxin
	APEAALDNTTNPTAY	MYA/13/2017 (Myanmar), PD287/2015 (India), IRN/72/2009 (Iran), BHU/3/2009 (Bhutan), INDIA 2013	0.2399	Non-antigen	Non-allergen	Non-toxin
	APEVALDNTTNPTAY	PD118/2017 (India), PD73/2017 (India)	0.3417	Non-antigen	Allergen	Non-toxin
	APEKALDNTTNPTAY	O/OMN/7/2001 (Oman)	0.1378	Non-antigen	Allergen	Non-toxin
	APETALENTTNPTAY	ISA/GRESIK (Indonesia), ISA/BANJARNEGARA (Indonesia), INDONESIA 2022, Depok 1, 2, Sukabumi 1, 2, Bandung 1, 3, 4, 5, 8, 9, Bogor 1, Palembang 1, 2, 3, 4, 6, 7, 8	0.3708	Non-antigen	Allergen	Non-toxin
133–150	NCKYGEGAVTNVRGDLQV	Strain O/ME-SA/Ind, MYA/13/2017 (Myanmar), PD287/2015 (India), O/OMN/7/2001 (Oman)	0.5798	Antigen	Allergen	Non-toxin
	NCRYGAGAVTNVRGDLQV	PD118/2017 (India), PD73/2017 (India)	0.7176	Antigen	Allergen	Non- toxin
	NCKYGESDVTNVRGDLQV	IRN/72/2009 (Iran), BHU/3/2009 (Bhutan)	0.7300	Antigen	Allergen	Non-toxin
	DCKYGESAVTNVRGDLQV	O/KUW/3/97 (Kuwait)	0.7244	Antigen	Allergen	Non-toxin
	NCKYGENNVPNVRGDLQV	INDIA 2013	0.6021	Antigen	Allergen	Non-toxin
	NCKYGEGAVANVRGDLQV	ISA/GRESIK (Indonesia), ISA/BANJARNEGARA (Indonesia), INDONESIA 2022, Depok 1, 2, Sukabumi 1, 2, Bandung 1, 3, 4, 5, 8, 9, Bogor 1, Palembang 1, 2, 3, 4, 6, 7, 8	0.5417	Antigen	Allergen	Non-toxin
192–205	AIHPEQARHKQKIV	Strain O/ME-SA/Ind, MYA/13/2017 (Myanmar), PD287/2015 (India), PD118/2017, PD73/2017 (India), ISA/GRESIK (Indonesia), ISA/BANJARNEGARA (Indonesia), INDONESIA 2022, Depok 1, 2, Sukabumi 1, 2, Bandung 1, 3, 4, 5, 8, 9, Bogor 1, Palembang 1, 2, 3, 4, 6, 7	0.6571	Antigen	Non-allergen	Non-toxin
	AIHPSQARHKQKIV	IRN/72/2009 (Iran), BHU/3/2009 (Bhutan)	0.7821	Antigen	Non-allergen	Non-toxin
	AIHPSEARHKQKIV	O/OMN/7/2001 (Oman), O/KUW/3/97 (Kuwait), INDIA 2013	0.8339	Antigen	Non-allergen	Non-toxin
	AIHPKQARHKQKIV	Palembang 8	0.7958	Antigen	Allergen	Non-toxin

### Prediction of *VP1* protein–ligand binding sites

**Figure 4:**
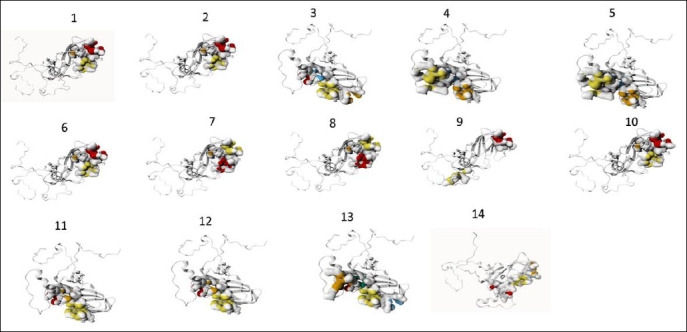
Ligand-binding site prediction and visualization of protein 3D structure using the PrankWeb server. Different colors indicate different pockets. Sample codes: strain O/ME_SA/Ind (1), MYA/13/2017 (2), PD287/2015 (3), PD118/2017 (4), PD73/2017 (5), IRN/72/2009, BHU/3/2009 (6), O/OMN/7/200 (7), O/KUW/3/97 (8), INDIA 2013 (9), ISA/GRESIK, ISA/BANJARNEGARA, INDONESIA 2022 (10), BANDUNG 1, 3, 4, 5, 9, DEPOK 1, BOGOR 1, PALEMBANG 1, 2, 3, 4, 6, 7, 9 (11), DEPOK 2, SUKABUMI 1, 2 (12), PALEMBANG 8 (13), BANDUNG 8 (14).

Protein–ligand binding site analysis of *VP1* revealed notable variation among isolates (Table 8). Palembang 8 exhibited the highest number of predicted binding pockets, enriched in threonine, tyrosine, glutamine, and asparagine residues capable of forming hydrogen bonds. Differences in pocket composition between ISA/Gresik/2022, ISA/Banjarnegara/2022, and the field samples suggested altered ligand-binding specificity. Structural visualization showed five high-probability binding pockets in Palembang 8, whereas other isolates displayed fewer than five pockets (Figure 4). Binding pockets ranked 1 or 2 were prioritized as the most probable functional sites.

**Table 8:** Analysis of the ligand-binding site of VP1 protein among collected samples and database.

No.	Sample	Pocket No.	Residues	Score	Rank
1	O/ME_SA/Ind	1	85_ASN, 87_THR, 98_LEU, 99_ASP, 101_THR, 105_THR, 168_LYS, 169_ALA, 170_THR	1.92	1
		2	50_VAL, 89_VAL, 90_PRO, 91_ASN, 92_GLY, 93_ALA, 163_ASN, 165_GLY, 166_ALA	1.79	2
		3	86_LEU, 88_TRP, 167_ILE	0.97	3
2	MYA/13/2017	1	85_ASN, 87_THR, 98_LEU, 99_ASP, 101_THR, 105_THR, 168_LYS, 169_ALA, 170_THR	1.92	1
		2	50_VAL, 89_VAL, 90_PRO, 91_ASN, 92_GLY, 93_ALA, 163_ASN, 165_GLY, 166_ALA	1.76	2
		3	78_VAL, 80_VAL, 86_LEU, 88_TRP, 115_LEU, 167_ILE	0.97	3
3	PD287/2015	1	71_TYR, 123_HIS, 125_VAL, 127_ALA, 130_TYR, 162_PHE	2.41	1
		2	50_VAL, 89_VAL, 90_PRO, 92_GLY, 93_ALA, 95_GLU, 98_LEU, 163_ASN, 165_GLY, 166_ALA	2.21	2
		3	86_ASN, 87_THR, 99_ASP, 101_THR, 168_LYS, 169_ALA, 170_THR	1.31	3
		4	52_ASP, 53_LEU, 72_TYR, 164_TYR	0.81	4
4	PD118/2017	1	71_TYR, 123_HIS, 125_VAL, 127_ALA, 130_TYR, 134_CYS, 160_PRO, 161_SER, 162_PHE	3.13	1
		2	132_GLY, 133_ASN, 135_ARG, 138_ALA, 141_VAL, 142_THR, 153_GLN, 156_ALA, 157_ARG, 158_THR	2.30	2
		3	50_VAL, 89_VAL, 90_PRO, 93_ALA, 95_GLU, 98_LEU, 163_LYS, 166_ALA	2.06	3
		4	52_ASP, 53_LEU, 72_TYR, 164_TYR	0.90	4
5	PD73/2017	1	71_TYR, 125_VAL, 127_ALA, 130_TYR, 134_CYS	3.13	1
		2	132_GLY, 133_ASN, 135_ARG, 138_ALA, 141_VAL, 142_THR, 153_GLN, 156_ALA, 157_ARG, 158_THR	2.30	2
		3	89_VAL, 90_PRO, 93_ALA, 95_GLU, 98_LEU, 163_LYS, 166_ALA	2.05	3
		4	52_ASP, 53_LEU, 72_TYR, 164_TYR	0.90	4
6	IRN/72/2009; BHU/3/2009	1	85_ASN, 87_THR, 98_LEU, 99_ASP, 101_THR, 105_THR, 168_LYS, 169_ALA, 170_THR	1.92	1
		2	50_VAL, 89_VAL, 90_PRO, 91_ASN, 92_GLY, 93_ALA, 163_ASN, 165_GLY, 166_ALA	1.72	2
		3	78_VAL, 80_VAL, 86_LEU, 88_TRP, 115_LEU, 167_ILE	0.98	3
7	O/OMN/7/2001	1	50_VAL, 89_VAL, 90_PRO, 91_ASN, 92_GLY, 93_ALA, 95_GLU, 163_ASN, 165_GLY, 166_ALA	2.35	1
		2	85_ASN, 87_THR, 98_LEU, 99_ASP, 101_THR, 105_THR, 168_LYS, 169_ALA, 170_THR	1.95	2
		3	78_VAL, 80_VAL, 86_LEU, 88_TRP, 115_LEU, 167_ILE	0.98	3
8	O/KUW/3/97	1	50_VAL, 89_VAL, 90_PRO, 91_ASN, 92_GLY, 93_ALA, 95_GLU, 163_ASN, 165_GKY, 166_ALA	2.26	1
		2	85_ASN, 87_THR, 98_LEU, 99_ASP, 101_THR, 105_THR, 168_LYS, 169_ALA, 170_THR	2.00	2
		3	78_VAL, 80_VAL, 86_LEU, 88_TRP, 115_LEU, 167_ILE	1.06	3
9	INDIA 2013	1	85_ASN, 87_THR, 98_LEU, 99_ASP, 100_ASN, 101_THR, 105_THR, 168_LYS, 169_ALA, 170_THR	1.71	1
		2	135_LYS, 139_ASN, 140_ASN, 156_ALA	1.36	2
10	INDONESIA 2022; ISA/GRESIK; ISA/BANJARNEGARA	1	85_ASN, 87_THR, 98_LEU, 99_GLU, 168_LYS, 169_ALA, 170_THR	2.11	1
		2	50_VAL, 89_VAL, 90_PRO, 91_ASN, 92_GLY, 93_ALA, 163_ASN, 165_GLY, 166_ALA	1.78	2
		3	78_VAL, 80_VAL, 86_LEU, 88_TRP, 115_LEU, 167_ILE	0.97	3
11	BANDUNG; DEPOK; BOGOR; PALEMBANG	1	71_TYR, 123_HIS, 125_VAL, 127_ALA, 130_TYR, 162_PHE	2.44	1
		2	50_VAL, 89_VAL, 90_PRO, 92_GLY, 93_ALA, 95_GLU, 98_LEU, 163_ASN, 165_GLY, 166_ALA	2.21	2
		3	52_ASP, 72_TYR, 164_TYR	0.82	3
12	DEPOK; SUKABUMI	1	71_TYR, 123_HIS, 125_VAL, 127_ALA, 130_TYR, 162_PHE	2.36	1
		2	50_VAL, 89_VAL, 90_PRO, 92_GLY, 93_ALA, 95_GLU, 98_LEU, 163_ASN, 165_GLY, 166_ALA	2.16	2
		3	52_ASP, 53_LEU, 72_TYR, 164_TYR	0.81	3
13	PALEMBANG 8	1	71_TYR, 123_HIS, 125_VAL, 127_ALA, 130_TYR, 162_PHE	2.38	1
		2	50_VAL, 89_VAL, 90_PRO, 92_GLY, 93_ALA, 95_GLU, 98_LEU, 163_ASN, 165_GLY, 166_ALA	2.18	2
		3	132_GLY, 133_ASN, 138_GLU, 153_GLN, 156_THR, 157_ARG, 158_ALA	2.10	3
		4	85_ASN, 87_THR, 99_GLU, 101_THR, 168_LYS, 169_ALA, 170_THR	1.51	4
		5	52_ASP, 53_LEU, 72_TYR, 164_TYR	0.79	5
14	BANDUNG 8	1	71_TYR, 122_PRO, 123_HIS, 125_VAL, 127_ALA, 130_TYR, 133_ASN, 134_CYS, 160_PRO, 161_THR, 162_SER, 163_PHE	3.19	1
		2	50_VAL, 89_VAL, 90_PRO, 92_GLY, 93_ALA, 95_GLU, 98_LEU, 164_ASN, 165_GLY, 167_ALA	2.27	2
		3	85_ASN, 87_THR, 99_GLU, 101_THR, 169_LYS, 170_ALA, 171_THR	1.58	3

### Molecular docking analysis between *VP1* and *TLR7*

**Figure 5:**
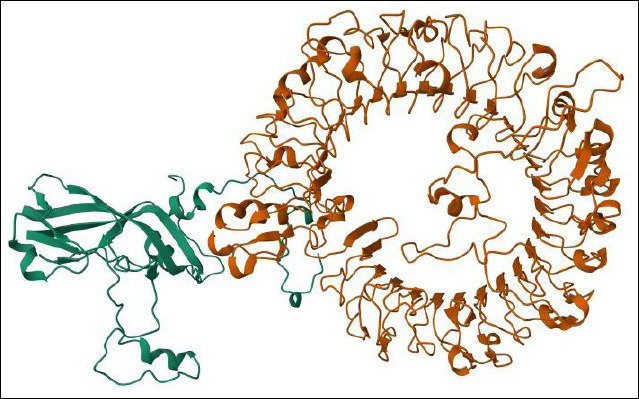
Visualization of docking between the VP1 protein and TLR7 using the HDOCK server, VP1 (green), TLR7 (orange).

Molecular docking analysis demonstrated variable interaction strength between *VP1* and *TLR7*. Palembang 8 exhibited the lowest docking score and highest confidence score, indicating the strongest predicted interaction with *TLR7* (Table 9). A more negative docking score reflected thermodynamically favorable binding, while confidence scores >0.7 indicated a high-probability of interaction; scores between 0.5 and 0.7 suggested possible binding, and values <0.5 indicated unlikely interactions. Palembang 8 formed a dominant docking cluster comprising 65 structures, indicating a stable and reliable binding mode. The associated Z-score (−1.2) confirmed significantly higher stability compared with other clusters. Energy decomposition analysis revealed strong electrostatic and van der Waals contributions, consistent with dense hydrogen-bonding networks and optimal shape complementarity. Residue-level interaction analysis further showed that Palembang 8 had the highest proportion of hydrogen bonds with *TLR7*, supporting its enhanced binding specificity (Table 10). Visualization of the *VP1*–*TLR7* complex confirmed that key binding epitopes were surface-exposed, facilitating effective receptor engagement (Figure 5).

**Table 9:** Prediction of interactions between VP1 protein and TLR7 using amino acid sequences from collected samples and database.

Sample	Docking score	Confidence score	RMSD	van der Waals energy	Electrostatic energy	Z-score	Cluster size
O/ME-SA/Ind	−286.07	0.9383	19.4 ± 0.1	−44.1 ± 4.6	−187.5 ± 19.1	−1.9	14
MYA/13/2017	−284.87	0.9369	13.3 ± 0.3	−49.3 ± 5.0	−208.8 ± 39.8	−2.4	4
PD287/2015	−320.49	0.9680	24.8 ± 0.4	−63.0 ± 8.9	−169.9 ± 25.6	−1.3	68
PD118/2017	−319.86	0.9676	0.8 ± 0.5	−70.9 ± 0.5	−128.5 ± 29.8	−1.7	5
PD73/2017	−311.30	0.9618	0.7 ± 0.4	−74.9 ± 3.5	−138.9 ± 23.4	−2.0	8
IRN/72/2009; BHU/3/2009	−292.93	0.9458	10.0 ± 0.5	−52.1 ± 11.4	−175.9 ± 23.5	−2.2	5
O/OMN/7/2001	−287.50	0.9399	17.0 ± 0.1	−42.9 ± 12.5	−206.1 ± 15.3	−1.5	4
O/KUW/3/97	−277.89	0.9281	14.8 ± 0.1	−49.8 ± 10.6	−174.2 ± 35.2	−2.3	4
INDIA 2013	−288.23	0.9407	10.7 ± 0.2	−49.8 ± 9.8	−138.1 ± 28.5	−1.1	6
INDONESIA 2022; ISA/GRESIK; ISA/BANJARNEGARA	−283.24	0.9349	12.0 ± 0.3	−45.9 ± 9.0	−315.8 ± 37.4	−2.4	7
BANDUNG 1, 3, 4, 5, 9; DEPOK 1; BOGOR 1; PALEMBANG 1, 2, 3, 4, 6, 7, 9	−287.37	0.9398	—	−74.3 ± 3.5	−135.7 ± 8.8	−1.7	11
DEPOK 2; SUKABUMI 1, 2	−319.04	0.9671	0.5 ± 0.3	−68.3 ± 8.4	−150.0 ± 27.1	−1.2	4
PALEMBANG 8	−324.27	0.9703	24.6 ± 0.1	−65.4 ± 6.7	−183.7 ± 18.3	−1.2	65
BANDUNG 8	−321.75	0.9688	24.8 ± 0.3	−64.9 ± 4.5	−168.6 ± 20.1	−1.4	76

RMSD = Root mean square deviation.

**Table 10:** Residue interactions between VP1 protein and TLR7 among collected samples and database.

Interaction parameter	A	B	C	D	E	F	G	H	I	J	K	L	M
Number of patients	50	50	38	39	31	53	53	55	45	40	39	44	38
Hydrogen bond (%)	24.0	22.0	23.7	17.9	9.7	22.6	18.9	18.2	20.5	20.5	17.9	25.0	21.1
Van der Waals (%)	66.0	68.0	55.3	61.5	64.5	64.2	71.7	72.7	70.5	71.8	64.1	59.1	60.5
π–π stack (%)	2.0	2.0	10.5	12.8	16.1	1.9	1.9	1.8	2.3	2.6	15.4	11.4	10.5
Ionic (%)	4.0	4.0	5.3	5.1	3.2	3.8	3.8	3.6	4.4	5.1	2.6	2.3	5.3
π-cation (%)	2.0	2.0	2.6	2.6	3.2	3.8	1.9	1.8	2.3	0.0	0.0	2.3	2.6

A = O/ME-SA/Ind, B = MYA/13/2017 (Myanmar), C = PD287/2015 (India), D = PD118/2017 (India), E = PD73/2017 (India), F = IRN/72/2009 (Iran), BHU/3/2009 (Bhutan), G = O/KUW/3/97 (Kuwait), H = INDIA 2013, I = ISA/1/2022, ISA/Gresik/2022, ISA/Banjarnegara/2022, J = Bandung 1, 3, 4, 5, 9, Depok 1, Bogor 1, Palembang 1, 2, 3, 4, 6, 7, 9, K = Depok 2, Sukabumi 1, Sukabumi 2, L = Palembang 8, M = Bandung 8, TLR7 = Toll-like receptor 7, VP1 = Viral protein 1, aa = Amino acid.

## DISCUSSION

### Persistence of FMD despite vaccination in Indonesia

FMD remains the most damaging viral disease affecting cattle production in Indonesia. Although a national FMD control program has been implemented using an inactivated vaccine containing serotype O strains O/Mya98/XJ/2010, O1 Campos, O1 Manisa, and IND/O/R2/75 (Decree of the Minister of Agriculture of the Republic of Indonesia No. 738/KPTS/PK.300/M/10/2022), FMD continues to circulate within the country. The vaccination campaign has reduced the frequency of clinical disease; however, unregulated animal movement facilitates the ongoing circulation of FMDV strains. In addition, Indonesia’s geographic position increases the risk of incursion of circulating strains from other Asian countries. Consequently, serotype O remains endemic and continues to circulate in Indonesia [6].

### Molecular diversity of capsid genes during the 2022–2023 outbreaks

This study presents the first molecular analysis of *VP1*, *VP2*, and *VP3* sequences from the 2022–2023 outbreaks in South Sumatra and West Java. The nucleotide identity of *VP1* decreased to 98.26–99.05% in several samples, while *VP3* identity declined to 98.48% in multiple isolates. These findings suggest ongoing antigenic drift that may influence vaccine matching. Phylogenetic analysis based on *VP1* nucleotide and amino acid sequences demonstrated that the collected samples formed clusters distinct from recent Indonesian reference isolates ISA/Banjarnegara/2022 and ISA/Gresik/2022. Specifically, three clusters were identified using nucleotide variation and two clusters using amino acid sequences. This pattern contrasts with previous reports in which 2022 Indonesian isolates clustered tightly within Ind-2001d or Mya-98 lineages, highlighting novel intra-country antigenic diversification within a single outbreak period. Multiple nucleotide and amino acid substitutions were detected relative to ISA/Banjarnegara/2022 and ISA/Gresik/2022, including changes at amino acid positions 4, 13, 129, 172, and 197 (Table 6). These substitutions are located near known immunogenic regions, including the G–H loop, B–C loop, and N-terminus of *VP1*, and may influence receptor binding and antigen recognition.

### Evolutionary mechanisms shaping FMDV diversity

The observed genetic heterogeneity of serotype O FMDV across different regions is likely driven by unrestricted livestock movement. Endemic co-circulation of multiple genotypes may facilitate recombination when closely related strains co-infect a host [17]. As an RNA virus lacking proofreading mechanisms, FMDV evolves rapidly, with mutation rates ranging from 10^-3^ to 10^-5^ substitutions per nucleotide site per year [18]. Each viral population exists as a quasispecies comprising genetically and antigenically diverse variants [19]. Because the principal immunogenic region of FMDV is located within the 1D region, *VP1* remains the most informative gene for investigating viral variation and evolutionary relationships among isolates [20].

### Implications for vaccine effectiveness and disease control

FMDV evolution predominantly occurs through point mutations, and the extent of genetic change influences viral adaptability, transmissibility, and pathogenicity [21, 22]. The emergence of new strains may compromise disease control in endemic regions when vaccine strains fail to provide adequate cross-protection [23]. Moreover, current vaccines do not prevent the establishment of a carrier state, as infected cattle and buffaloes may excrete the virus for extended periods regardless of vaccination status. Although mortality is uncommon, FMD causes severe disease, and vaccination primarily reduces clinical severity rather than preventing infection or halting transmission.

### Immunoinformatic insights into *VP1* epitope variation

Epitope prediction analysis of *VP1* revealed variation in peptide binding to host T- and B-cell receptors among the collected samples compared with database sequences. All predicted peptides were antigenic and non-toxic, although some showed potential allergenicity based on in silico analysis. The BLV envelope protein (UniProt: P03380) served as a positive control and was predicted to be antigenic, non-allergenic, and non-toxic, supporting the robustness of the analytical pipeline. Certain isolates exhibited unique T-cell binding shifts, such as BoLA-T7–associated changes linked to A4T and A13T substitutions. Given the genetic diversity of Indonesian cattle populations, allele-specific immune responses may influence vaccine performance at regional levels. These findings indicate host-allele-dependent immunogenic divergence, which has not been previously reported for Indonesian FMD outbreaks. Key B-cell antigenic sites of FMDV are located within surface-exposed structural motifs of the capsid, including the N-terminus, B–C, E–F, F–G, G–H loops, and the C-terminus [24]. The conserved RGD motif within the G–H loop of *VP1* is essential for integrin-mediated viral entry [25], although linear peptides spanning this region provide limited protection in natural hosts [26]. While neutralizing antibodies are central to protective immunity, T-cell responses are also required for robust immune protection [27], mediated through peptide presentation by MHC molecules to T-cell receptors [28].

### Structural and functional relevance of *VP1* binding sites

Previous epitope studies of serotype O have primarily focused on neutralizing domains of *VP1* at amino acid positions 133, 143–144, 147–149, 151–153, 161, and 164–167 [29], as well as positions 50 and 52 [30] and 199–202 [31]. Computational analyses have predicted multiple B- and T-cell epitopes for serotypes A and O [32], while secondary structure and antigenicity profiling of *VP1* has further identified dominant B-cell epitopes [33, 34]. In the present study, ligand-binding pocket analysis revealed notable differences in binding specificity among Indonesian isolates. Palembang 8 exhibited the highest number of binding pockets, enriched with threonine, tyrosine, glutamine, and asparagine residues, distinguishing it from ISA/2022 and other reference isolates. These findings represent the first structural binding-pocket mapping of Indonesian FMDV serotype O isolates.

### Molecular docking of *VP1* with *TLR7* and study limitations

Protein–protein docking analysis demonstrated that Palembang 8 showed the lowest docking and confidence scores for interaction between *VP1* and *TLR7*, indicating a higher probability of binding [39]. This isolate exhibited extensive hydrogen-bonding, strong electrostatic and van der Waals interactions, and high structural complementarity, suggesting enhanced engagement with innate immune receptors. To our knowledge, this is the first report describing the *VP1*–*TLR7* binding potential of FMDV isolates, providing new insights into innate immune recognition mechanisms. Nevertheless, this study is limited by its reliance on in silico predictions without experimental neutralization assays or empirical antigenicity testing. Future work should incorporate wet-lab validation, refined structural modeling, and epitope conservancy monitoring to strengthen these findings.

## CONCLUSION

This study provides comprehensive molecular, immunoinformatic, and structural evidence that FMDV serotype O circulating in Indonesia during the 2022–2023 outbreaks is undergoing active genetic and antigenic diversification. Sequence analysis demonstrated reduced nucleotide identity in *VP1* (98.26–99.05%) and *VP3* (down to 98.48%) compared with recent Indonesian reference isolates. Phylogenetic reconstruction revealed three nucleotide-based clusters and two amino acid–based clusters, indicating intra-country diversification distinct from previously reported tight clustering within Ind-2001d or Mya-98 lineages. Multiple novel amino acid substitutions in *VP1* (positions 4, 13, 129, 172, and 197) were identified near key immunogenic regions, including the G–H loop, B–C loop, and N-terminus. Immunoinformatic analyses showed isolate-specific variation in T- and B-cell epitope binding, including BoLA-T7–associated shifts, while structural analyses identified isolate-dependent variation in *VP1* ligand-binding pockets and enhanced *VP1*–*TLR7* interaction potential, particularly in Palembang 8.

These findings have direct implications for FMD control in Indonesia. The observed antigenic drift in *VP1* suggests that current vaccine strains may gradually lose matching efficiency against emerging local variants. Host-allele-dependent epitope variation further indicates that vaccine responsiveness may differ among cattle populations. The identification of isolates with enhanced *TLR7* interaction potential highlights the relevance of innate immune engagement in shaping host responses and may inform future vaccine adjuvant or immunogen design. Continuous molecular surveillance integrating phylogenetics and immunoinformatics is therefore essential to support adaptive vaccine updating and region-specific control strategies.

The major strength of this work lies in its integrated approach, combining molecular sequencing, phylogenetic analysis, epitope prediction, antigenicity/allergenicity/toxicity screening, structural pocket mapping, and protein–protein docking. This multi-layered framework enabled functional interpretation of genetic variation beyond descriptive phylogeny and represents the first report linking *VP1* sequence diversity with predicted *TLR7* binding potential in Indonesian FMDV isolates.

This study is limited by its reliance on in silico analyses without experimental validation. Neutralization assays, empirical antigenicity testing, and functional immune assays were not performed. In addition, the number of isolates analyzed, although representative, may not capture the full national diversity of circulating FMDV serotype O.

Future studies should include wet-lab validation of predicted epitopes, vaccine cross-neutralization assays, and longitudinal surveillance to monitor epitope conservancy. Structural refinement using higher-resolution models and experimental confirmation of *VP1*–*TLR7* interactions will further strengthen mechanistic understanding. Expanding analyses to additional provinces and integrating host genetic data may also clarify host–virus co-evolution dynamics.

Overall, this study demonstrates that FMDV serotype O in Indonesia is evolving through measurable genetic, antigenic, and structural changes with potential consequences for vaccine effectiveness and immune recognition. The findings underscore the need for continuous, integrative surveillance and evidence-based vaccine adaptation to sustain effective FMD control in endemic settings.

## DATA AVAILABILITY

The data supporting this study are included in the manuscript. All full sequence VP1 data were deposited in GeneBank with accession numbers PX406137-PX406155.

## AUTHORS’ CONTRIBUTIONS

RIA1, ATW, RIA2, NLPIM, and TPP: Drafted the manuscript. RIA1, SS, TPP, and HR: Collected the sample and performed data analysis. RIA1: Extracted RNA, primer design, and gene amplification. RIA1 and TP: Performed epitope and docking analysis. ATW, RIA2, NLPIM, and TPP: Methodology and software validation. All authors have read and approved the final version of the manuscript.
